# A prospective analysis of thulium laser enucleation in benign prostatic hyperplasia comparing low- and high-power approaches for prostates exceeding 80 g

**DOI:** 10.1007/s00345-024-04901-w

**Published:** 2024-04-27

**Authors:** Ahmed Y. Abdelaziz, Islam Kamal, Mahmoud A. Abdelhakim, Mostafa Abdelmohsen, Alaa Meshref, Islam Naser, Samer Morsy

**Affiliations:** Urology Department, Cairo University, Cairo, Egypt

**Keywords:** Thulium laser, Enucleation, High power, Low power

## Abstract

**Introduction and objectives:**

To compare the perioperative and functional outcomes of low-power and high-power thulium:YAG VapoEnucleation (ThuVEP) of the prostate for the treatment of large-volume benign prostatic hyperplasia (BPH) (> 80 ml).

**Patients and methods:**

A prospective analysis of 80 patients with symptomatic BPO and prostatic enlargement (more than 80 ml) was conducted. They were divided randomly into two groups (40 patients in each group). One group was treated with low-power ThuVEP, and the other group was treated with high-power ThuVEP.

All patients were assessed preoperatively and early postoperatively, and 12-month follow-up data were analyzed. The complications were noted and classified according to the modified Clavien classification system.

**Results:**

The mean age at surgery was 68 (± 6.1) years, and the mean prostate volume was 112 (± 20.1) cc, and there were no differences between the groups (*p* = 0.457). The mean operative time was 88.4 ± 11.79 min for group A and 93.4 ± 16.34 min for group B, while the mean enucleation time was 59.68 ± 7.24 min for group A and 63.13 ± 10.75 min for group B. There were no significant differences between the groups regarding catheterization time and postoperative stay. The quality of life (QoL), International Prostate Symptom Score (IPSS), maximum urinary flow rate (Qmax), postvoiding residual urine (PVR), and prostate volume improved significantly after treatment and were not significantly different between those treated with the different energies. The incidence of complications was low and did not differ between both the groups.

**Conclusion:**

Low-power ThuVEP is feasible, safe, and effective with comparable results with high-power ThuVEP in the treatment of BPO.

## Introduction

Thulium laser (TmL) was first used by Xia et al. in 2005 for prostate resection [1]. Since that time, many advantages have been attributed to thulium laser used for both stone lithotripsy and tissue purposes. Besides the shallow penetration depth (0.2 mm) [2], TmL provides a continuous-wave pattern and, consequently, an easier-to-learn prostate enucleation technique with a shorter learning curve in comparison to holmium laser enucleation of the prostate (HoLEP) [3]. Although thulium has good hemostatic property, factors other than the energy used may affect intraoperative hemostasis and postoperative bleeding [4].

It was not until Pariser and his colleagues utilized a high-power thulium (150 W) in 2014 for prostate vaporization in a short outcome series, and before that time, the majority of studies had been performed with an upper-limit power of 120 W [5]. Previous studies showed that high-power thulium laser, typically exceeding 100 W, provides higher tissue ablation rates and can be chosen in situations necessitating expedited surgical times or larger prostates [6, 7]. Meanwhile, low-power thulium laser ranging from 30 to 60 W is associated with precise tissue ablation and minimal thermal damage to surrounding structures [8].

On the contrary, low-power thulium laser (40 W) has been studied in the literature for enucleation purposes using the different aforementioned techniques [9–11]. Different thulium energy approaches either ThuLEP, pulsed-wave thulium fiber laser enucleation of the prostate (PW-ThuFLEP), or continuous-wave thulium fiber laser enucleation of the prostate (CW-ThuFLEP) revealed no significant differences as regards perioperative and clinical outcomes [12, 13]. Therefore, this study was conducted to compare the enucleation efficiency of high-power and low-power thulium vapoenucleation (ThuVEP) for large prostatic adenomas as a primary endpoint. The secondary endpoints were to assess and to compare the early and delayed complications related to each power of TmL.

## Patient and methods

This was a prospective randomized study conducted at Kasr Alainy Hospital, Cairo University Tertiary Center, between January 2020 and January 2022 including all patients with large prostate (prostate ˃ 80 g) indicated for surgical intervention (maximal flow rate, Qmax, less than 15 mL/s or international prostate symptom score, IPSS, ≥ 18 or with recurrent attacks of urine retention refractory to medical treatment). However, patients with prostate cancer, concomitant bladder stones, urethral stricture, and urodynamically diagnosed detrusor underactivity were excluded. The study was approbated by the local committee of ethics with IRB number MD-189-2020, and written consent was obtained from all participants. The thulium laser unit used in this study was the Revolix DUO® (Lisa laser, Katlenburg-Lindau, Germany), equipped with a 550 μm RigiFib also from Lisa Laser in Katlenburg-Lindau, Germany. Patients were randomized according to computer-generated block randomization. Power settings were 100 W for enucleation and 80 W for coagulation in the high-power group (group A) and 30 W for enucleation and 25 W for coagulation in the low-power group (group B). The resectoscope was a 26 Ch caliber with continuous irrigation (Karl Storz, Tuttlingen, Germany), and the morcellation was accomplished by Storz morcellator (Karl Storz GmbH & Co., Tuttlingen, Germany) which was inserted by means of a nephroscope sheath in all procedures. The early release en bloc enucleation technique as described by Saitta et al. [14] was carried out by two surgeons with 5 years prior experience of prostate enucleation (40–50 cases per year). All procedures were carried out using normal saline when the patients were under spinal anesthesia. At the end of the procedure, a 22 F three-way urethral catheter was fixed with continuous bladder irrigation by normal saline. The irrigation was stopped the next morning based on standard department protocol. We removed the urethral catheter on the second postoperative day unless there was gross hematuria and the patients were discharged after being able to void adequately. All patients received perioperative antibiotics in the form of the second-generation cephalosporin.

All patients were assessed through full medical history, surgical history, Qmax, postvoiding residual urine (PVR), IPSS, IIEF5 questionnaires, and routine preoperative laboratories along with serum PSA level. The prostate size was measured through trans-rectal ultrasound, while the prostate needle biopsy and urodynamic testing were performed only if indicated.

The primary outcome of the study was the enucleation efficiency, determined by the ratio of the resected weight of the prostate to the enucleation time (from the insertion of the laser fiber until removal), expressed in grams per minute. Secondary outcomes included various measures such as operative efficiency (the ratio of resected prostate weight to operative time in grams per minute, laser rate (calculated as laser energy divided by enucleation time), and the percentage of resected tissue.

The intraoperative parameters such as the enucleation time, total operative time, and morcellation time were monitored and compared between both the groups. The intraoperative complications according to the Clavien–Dindo classification such as subtrigonal dissection, capsular perforation, and bladder injury during morcellation and the need for blood transfusion were also documented. During the hospital stay, postoperative clot urine retention, fever, need for auxiliary hemostatic procedures, mean catheterization time, and hospital stay were reported.

All patients were asked to visit the outpatient clinic after the first week of catheter removal to assess the act of micturition and the early postoperative complications and then after 1, 3, 6, and 12 months. During the follow-up, Qmax, PVR, IPSS, IIEF5, quality of life index (QOL), and PSA were measured according to the scheduled regimen, and the postoperative mean values were compared to the preoperative values in each group and between both the groups. Additionally, the postoperative complications such as persistence of obstructive symptoms, urine retention requiring catheterization, bladder neck contracture, and urethral stricture were documented and compared between both the groups.

## Statistical analysis

The paired t-test was employed for numerical data matching, given the sufficiently large sample size, while the McNemar’s test was utilized for comparing categorical data. In the examination of the general linear model, repeated-measures ANOVA was applied for all comparisons involving two variables across time among more than three time points. IBM SPSS (Statistical Package for the Social Science; IBM Corp., Armonk, NY, USA) version twenty-two for Microsoft Windows was used for all statistical computations.

## Results

**Fig. 1:**
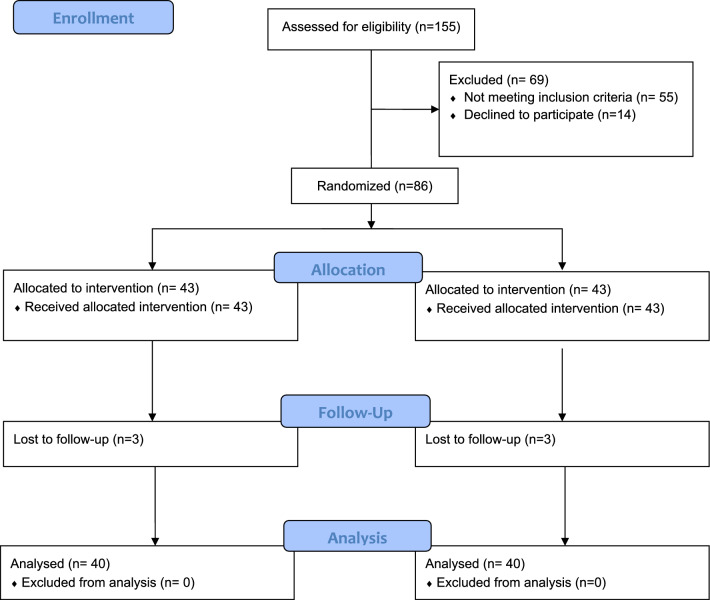
**Consort figure:** The total number of eligible patients and the number of analyzed patients at the end of the study

One hundred and fifty-five patients were screened, and only 86 patients met the necessary criteria and chose to enroll in the study. Six patients were also excluded due to missed follow-up, and eventually, 40 patients for each group underwent the procedure according to the preoperative randomization and conformed to the follow-up regimen (Fig. 1).

There was no statistically significant difference between both the groups regarding the preoperative parameters such as age, prostate size, PSA level, Qmax, PVR, IPSS, and the indication for surgery (*P* value ˃ 0.005). Thirteen patients were on ongoing antiplatelet/anticoagulant therapy (seven patients in group A and six patients in group B) (Tables 1, 2).

**Table 1:** Perioperative data

	Group A (*n* = 40)	Group B (*n* = 40)	*P* value
Age	68.63 ± 4.92	68.40 ± 6.18	0.85
*Presentation*			
Refractory retention	11 (27.5%)	8 (20%)	0.789
Recurrent hematuria	4 (10%)	4 (10%)	
High IPSS	25 (62.5%)	28 (70%)	
*Comorbidities*			
Diabetes mellitus (DM)	13 (32.5%)	14 (35%)	0.982
Hypertension	18 (45%)	13 (32.5%)	
Ischemic heart disease (IHD)	4 (10%)	5 (12.5%)	
Antiplatelet therapy	7(17.5%)	6 (15%)	
*Operative data*			
Operative time	88.40 ± 11.79 min	93.40 ± 16.34 min	0.12
Enucleation time	59.68 ± 7.24 min	63.13 ± 10.75 min	0.30
Morcellation time	19.60 ± 5.70 min	20.40 ± 6.80 min	0.142
Resected weight	105.38 ± 12.94	100.98 ± 13.54	0.90
Enucleation efficiency	1.76 ± 0.25	1.60 ± 0.55	0.60
Operation efficiency	1.02 ± 0.30	1.08 ± 0.20	0.60
Laser energy	154.75 ± 22.71 kJ	86.3 ± 17.39 kJ	< 0.001
Hospital stay	22 ± 3.4 h	19 ± 1.7 h	0.101
Catheterization duration	15.5 ± 2.5 h	16.6 ± 2 h	0.33
Preoperative Hb	13.2 ± 0.6 g/dl	12.9 ± 0.9 g/dl	0.873
Postoperative Hb	12.6 ± 0.5 g/dl	12.2 ± 0.4 g/dl	0.910
Hb drop	0.7 ± 0.2 g/dl	0.6 ± 0.4 g/dl	0.861

**Table 2:** Preoperative and postoperative BOO assessment

	Group A	Group B	*P* value
	Mean ± SD (*n* = 40)	Mean ± SD (*n* = 40)	(A vs. B)
Qmax (pre) (mL/s)	7.60 ± 1.98	6.91 ± 2.10	0.195
Qmax (post 1 month)	25.3 ± 11.3	27 ± 9.3	0.921
Qmax (post 3 months)	29.2 ± 7.3	28 ± 10.3	0.728
Qmax (post 6 months)	30 ± 5.3	31 ± 4.1	0.852
Qmax (post 12 months)	26 ± 6.1	25.3 ± 9.3	0.123
*P* value	< 0.004	< 0.0023	
PVR (pre) (mL)	129.97 ± 23.86	126.81 ± 19.85	0.576
PVR (post 1 month)	30 ± 11.2	27.4 ± 9.4	0.672
PVR (post 3 months)	25.5 ± 9.7	26 ± 7.3	0.729
PVR (post 6 months)	27.5 ± 3	25 ± 2.2	0.832
PVR (post 12 months)	22 ± 4.2	23.2 ± 3.5	0.932
*P* value	< 0.002	< 0.003	
IPSS (pre)	26 ± 1.95	25.69 ± 2.15	0.551
IPSS (post 1 month)	13.2 ± 3.2	8.1 ± 2.4	0.003
IPSS (post 3 months)	12 ± 2.1	7.2 ± 1.5	0.0041
IPSS (post 6 months)	7.32 ± 3	6.4 ± 2.4	0.062
IPSS (post 12 months)	5 ± 2.1	5.2 ± 2.9	0.321
*P* value	< 0.004	< 0.002	
IIEF-5 (pre)	15.13 ± 1.67	15.05 ± 1.93	0.853
IIEF-5 (post 3 months)	15 ± 1.92	14.5 ± 1.52	0.732
IIEF-5 (post 6 months)	16.3 ± 1.3	16 ± 1.72	0.723
IIEF-5 (post 12 months)	16 ± 1.32	15.3 ± 1.42	0.832
*P* value	> 0.06	> 0.08	
Prostate size (pre)	115.33 ± 20.19	111.90 ± 20.15	0.450
Prostate size (post)	20.75 ± 5.33	20 ± 5.10	1
Total PSA (pre) (ng/ml)	4.57 ± 1.31	4.52 ± 1.36	0.861
PSA (post 6 months)	1.24 ± 0.37	1.06 ± 0.31	0.052
PSA (post 12 months)	1.1 ± 0.12	1.04 ± 0.41	0.061
QOL (pre)	4.07 ± 1.00	4.13 ± 0.91	0.819
QOL (post)	1.67 ± 0.78	1.53 ± 0.62	0.732
*P* value	0.003	0.0029	

The mean operative time was 88.4 ± 11.79 min for group A and 93.4 ± 16.34 min for group B, while the mean enucleation time was 59.68 ± 7.24 min for group A and 63.13 ± 10.75 min for group B. There were no statistically substantial differences between both the groups regarding the mean operative time (*P* value = 0.12), morcellation time (*P* value = 0.3), enucleation efficiency (resected weight of the prostate divided by enucleation time) (*P* value = 0.6), or operation efficiency (*P* value = 0.6). Operative parameters were similar between the two groups (Table 1).

The mean used laser energy was 86.3 ± 17.39 kJ in the low-power group and 154.75 ± 22.71 kJ in the high-power group (*P* = < 0.001). The mean laser rate (laser energy consumed divided by enucleation time) was 1.26 ± 0.21 and 2.43 ± 0.18 kJ/min (P = < 0.001).

The black eschars were observed after complete enucleation and hemostasis in 22 patients (55%) in group A and 6 patients (15%) in group B without a significant impact on the endoscopic visualization in both the groups. Moreover, there were no statistically significant differences between both the groups regarding the need for blood transfusion represented by hemoglobin drop (one patient only for each group) and intraoperative complications (two patients in group A had a mucosal bladder injury, and three patients in group B and one patient from each group had a minor subtrigonal dissection) (*P* value ˃ 0.005) (Table 3).

**Table 3:** Early and delayed complications

Grade	Group A (*n* = 40)	Group B (*n* = 40)	*P* value
*Grade 1*			
Subtrigonal dissection	1(2.5%)	1 (2.5%)	0.921
Bladder mucosal injury	2 (5%)	3 (7.5%)	0.821
Capsule perforation	0	0	
Clot retention of urine	1 (2.5%)	0	0.9
Transient irritative symptoms (dysuria)	20 (50%)	6 (15%)	0.001
Transient stress incontinence	3 (7.5%)	2 (5%)	0.132
*Grade 2*			
UTI	1 (2.5%)	2 (5%)	0.235
Blood transfusion	1(2.5%)	1(2.5%)	0.9
*Grade 3*			
Bladder neck contracture	1(1.8%)	0	0.951
Urethral stricture	0	0	

No statistically significant difference was observed between both the groups concerning hospital stay, time of catheter removal, and enucleated prostate volume (*P* value ˃ 0.005). There was only one patient who developed clot urine retention in group A for which clot evacuation was done and bladder irrigation was recontinued without the need for hemostatic procedures (Tables 2, 3).

All patients could void freely after catheter removal, and there was a statistically significant improvement in each group between the preoperative and postoperative mean Qmax, PVR, and QOL score at 1, 3, 6, and 12 months, but it was not significant when comparing both the groups (*P* value ˃ 0.005) (Table 2). However, the irritative symptoms (namely urgency and dysuria) were statistically different between both the groups in the first 3 months (50% in the high-power group versus 15% in the low-power group) (*P* value: 0.001); it did not last for 6 and 12 months (see Tables 2, 3). There was no substantial difference between both the groups regarding the delayed complications (bladder neck contracture and urethral stricture) (Table 3). Histopathology of prostatic tissue showed BPH in all cases.

## Discussion

Since described by Scoffone et al., the early release enucleation technique has gained a worldwide popularity [15]. Thereafter, some modifications to the prime technique have been made, as suggested by many authors [14], to make the procedure easier to learn and to achieve better functional outcomes.

In the present study, there was a substantial statistical difference between both the groups regarding the transient irritative symptoms following the procedure, but there were no significant differences regarding the total operative time, hospitalization time, and mean catheterization time, also in early and delayed complications.

During prostate enucleation, extensive energy consumption could lead to tissue carbonization “black escharing” which might obscure the surgical planes and cause harm to the adjacent capsule and urethral sphincter [16]. Therefore, mechanical dissection-dependent enucleation using different lasers has been advocated by some authors to avoid loss of surgical planes, whereas the prostate enucleation relies mainly on the mechanical dissection exerted through the resectoscope sheath tip after making the initial incisions [17–19]. Following the aforementioned technique during the study, these black eschars were detected optically in 55% of group A and 15% of group B, albeit without a significant impact on the surgical planes in both the groups.

In an ex vivo experimental study, Huusmann et al. pointed out that the laser damage zone for thulium (continuous and pulsed) and holmium lasers is almost similar except for the 5-W Tm laser [20]. The authors also looked into the penetration depth—increasing laser power correlation—and found that more laser power could bring about more penetration depth and related laser damage zone, though it is highly controllable, especially with pulsed TmL which creates less carbonization than the continuous-wave TmL. In another in vitro experimental study, Hein and his colleagues attempted to assess the thermal effect of TmL and concluded that adverse thermal injury could be reached especially with high-power laser and low irrigation fluid volume and the generated heat could invade the prostate tissue with a potential harm to the nearby neurovascular bundles [21]. However, in consistence with the present study, Dmitry Enikeev and his colleagues found, through a clinical trial comparing thulium laser prostate vaporization (using 120 W) and conventional monopolar TURP, that TmL vaporization could preserve or even improve the erectile function after successful bladder outlet reduction [22].

Post-laser prostate enucleation irritative symptoms have been described by many studies. The pooled calculated incidence of such symptoms in a meta-analysis of eight studies reached up to 9% of patients who underwent the procedure [23]. Such symptoms, even transient in the majority of cases, may have a negative impact on the patients’ quality of life, and the management is still under debate [24]. A true explanation of these symptoms has not been established, but laser-induced capsular irritation along with urinary tract infections has been suggested [23]. Relatedly, in this study, the high-power group manifested much more irritative symptoms than the low-power group in the first three months, and these symptoms did not last for 6 months after the procedure.

Omar and his colleagues found that low-power thulium enucleation demonstrated a secure and effective outcome, obviating the necessity for a high-power thulium laser device [10]. In our previous study evaluating the surgical outcomes of low-power ThuLEP, we concluded that low-power ThuLEP proves to be a valuable therapeutic option, effectively treating patients with enlarged prostates and yielding satisfactory outcomes for both urinary and sexual functions [8].

Prior research extensively assessed the comparison between low-power and high-power HoLEP, demonstrating the non-inferiority of low-power HoLEP as regards perioperative parameters and functional outcomes [25]. Scoffone [26] in his systemic meta-analysis to assess the safety of low-power HoLEP in BPH and Gkolezakis and his colleagues [27] proved that the utilization of low-power HoLEP may be feasible, secure, and efficacious, potentially contributing significantly to diminishing the occurrence, severity, and duration of postoperative dysuria.

We believe that in addition to the efficacy and safety of using low-power ThuLEP, there might be a potential decrease in the initial cost of the laser procedure when utilizing a low-power machine and eliminating the necessity for high-current sockets which are not typically installed in operating rooms. Additionally, the diminished heat generation associated with low-power machines results in reduced demands on the air-conditioning system.

To the best of our knowledge, this is the first prospective study to evaluate the safety and efficacy of low-power ThuLEP in direct comparison with high-power ThuLEP. Despite being a randomized prospective study, this study is limited by the small sample size and additional comparative studies are essential to validate the efficacy of low-power ThuLEP across various enucleation techniques. While affirming the validity of the physical background for low-power ThuLEP, this study advocates for its utilization, particularly encouraging surgeons equipped with low-power machines to adopt this method.

## Conclusion

Both high-power and low-power thulium laser enucleation could provide comparable results apropos bladder outlet obstruction due to BPH.

## Data Availability

Not applicable.
